# Porcine Interleukin-2, IL-4 and IL-6 Combined with a Colloidal Manganese Adjuvant Enhance PCV2-Mhp Bivalent Inactivated Vaccine Immunogenicity in Mice

**DOI:** 10.3390/biology15141163

**Published:** 2026-07-16

**Authors:** Junjie Peng, Linhan Zhang, Dafang He, Gang Wang, Jianglin Li, Shanshan Zhu, Rong Gao

**Affiliations:** 1 National Engineering Research Center for Biomaterial, Sichuan University, Chengdu 610065, China; pengjunjie93@163.com (J.P.); wgang@scu.edu.cn (G.W.); 2 Key Laboratory of Bio-Resource and Eco-Environment of Ministry of Education, College of Life Sciences, Sichuan University, Chengdu 610065, China; linhanzhang@outlook.com (L.Z.); cxandhdfang@163.com (D.H.); 3 Sichuan Sanyoukang Biotechnological Co., Ltd., Chengdu 610093, China; 4 Sichuan Academy of Animal Science, Chengdu 610066, China; yujiang1465@126.com; 5 Sichuan University Library, Sichuan University, Chengdu 610065, China

**Keywords:** PCV2, Mycoplasma hyopneumoniae, bivalent inactivated vaccine, IL-2, IL-4, IL-6, MnJ(beta), cytokine adjuvant, murine model

## Abstract

Porcine circovirus type 2 (PCV2) and Mycoplasma hyopneumoniae (Mhp) are major contributors to the porcine respiratory disease complex. Although PCV2-Mhp bivalent inactivated vaccines are useful for simultaneous disease control, their immunogenicity may be improved by adjuvant optimization. This study evaluated a composite adjuvant consisting of porcine interleukin-2 (IL-2), IL-4, IL-6 and MnJ(beta), a colloidal manganese adjuvant, in an initial murine immunogenicity model. Thirty female Kunming mice were assigned to three groups (*n* = 10/group): bivalent antigen plus IL-2/IL-4/IL-6/MnJ(beta), bivalent antigen plus MnJ(beta), or phosphate-buffered saline. Body weight, complete blood count, peripheral blood T- and B-cell subsets, PCV2-specific IgG and Mhp-specific indirect hemagglutination titers were monitored after primary and booster immunization. The composite formulation did not suppress body-weight gain or induce sustained abnormalities in erythrocyte- or platelet-related indices. WBC, neutrophil, lymphocyte and monocyte counts were elevated in group A at days 7 and 28 post-primary immunization, indicating transient immune activation. Day-56 flow cytometry indicated increased CD19+IgM-IgD- B-cell and effector/memory T-cell-associated responses. PCV2-specific IgG increased from day 14 onward. At day 56, the OD450 value in group A reached 1.532 ± 0.006, compared with 1.095 ± 0.004 in group C1 and 0.102 ± 0.002 in group C2, corresponding to approximately 1.40-fold and 15.09-fold higher levels than the MnJ(beta)-adjuvanted and PBS control groups, respectively. Mhp-specific IHA titers were also maintained at high levels after booster immunization; at day 56, group A showed a log2 endpoint titer of 13.00 ± 0.00, corresponding to a GMT of 1:8192, whereas group C1 showed a log2 endpoint titer of 12.00 ± 0.00, corresponding to a GMT of 1:4096, and group C2 remained negative. These results indicate that the IL-2/IL-4/IL-6/MnJ(beta) composite adjuvant demonstrates potential for improving antibody and peripheral lymphocyte responses to PCV2-Mhp bivalent antigen, but protective efficacy must be confirmed in target-species challenge studies.

## Simple Summary

Porcine circovirus type 2 (PCV2) and Mycoplasma hyopneumoniae (Mhp) are economically important respiratory pathogens of pigs. Improved adjuvant strategies are needed for bivalent inactivated vaccines, but early-stage screening must be interpreted cautiously when performed outside the target species. In this study, we evaluated a composite adjuvant containing porcine IL-2, IL-4 and IL-6 together with a colloidal manganese adjuvant, MnJ(beta), in a 56-day Kunming mouse immunogenicity model. The formulation did not suppress body-weight gain and did not cause sustained abnormalities in erythrocyte- or platelet-related hematological indices, although transient leukocyte, neutrophil, lymphocyte and monocyte increases were observed after immunization. Compared with MnJ(beta)-adjuvanted antigen alone, the composite formulation increased PCV2-specific IgG responses, maintained Mhp-specific IHA titers during the observation period, and promoted peripheral B-cell and effector/memory T-cell phenotypes at day 56. These findings support further target-species evaluation of this cytokine/manganese adjuvant strategy, including expanded safety assessments and PCV2/Mhp challenge studies in piglets.

## 1. Introduction

Porcine circovirus type 2 (PCV2), a small, non-enveloped, single-stranded DNA virus, persists as a significant pathogen associated with porcine circovirus diseases and porcine respiratory disease complex (PRDC) [1,2,3]. Epidemiological data from China reveal ongoing widespread PCV2 circulation, with genotype shifts and heterogeneous herd-level infections complicating vaccine matching and long-term disease control [4,5,6]. Worldwide reports over the past decade also indicate substantial genotype diversity and continued evolution of PCV2, supporting the need for broadly immunogenic and adaptable vaccine strategies [7]. Mycoplasma hyopneumoniae (Mhp), the causative agent of enzootic pneumonia, colonizes the respiratory ciliated epithelium, impairs mucociliary clearance, and increases susceptibility to secondary bacterial and viral infections [8,9]. Given their frequent co-occurrence in modern swine production systems and potential combined effects on respiratory disease severity, enhanced PCV2-Mhp bivalent vaccination strategies remain crucial for PRDC management [3,10,11].

Vaccination currently represents the most practical intervention for reducing PCV2- and Mhp-related disease burden. While commercial PCV2 vaccines can mitigate clinical disease, viremia, shedding, and mortality, and Mhp vaccination may reduce lung lesions and performance losses, existing vaccines often fail to completely prevent infection, colonization, or transmission [10,11,12]. Emerging research on PCV2 genotype coverage, intradermal immunization, and ready-to-use PCV2-Mhp vaccine formulations indicates that efficacy depends on antigenic breadth, delivery route, vaccine platform, and the quality of induced cellular immunity [13,14,15,16,17,18]. Consequently, safe and effective adjuvants remain essential for enhancing the magnitude, quality, and durability of immune responses to multivalent or bivalent inactivated vaccines.

Cytokine-based immunomodulators have gained attention as vaccine adjuvants due to their ability to shape adaptive immune responses. IL-2 promotes T-cell activation, clonal expansion, and cytotoxic lymphocyte function while enhancing B-cell responses [19]. IL-4 facilitates B-cell maturation, immunoglobulin class switching, and Th2-associated immunity [20], whereas IL-6 participates in acute-phase responses, B-cell differentiation, antibody production, and T-cell activation [21]. These complementary functions position IL-2, IL-4, and IL-6 as promising candidates for balancing cellular and humoral immunity when combined with conventional vaccine antigens and innate immune adjuvants [22,23]. A previous piglet study demonstrated that a chitosan nanoparticle-encapsulated recombinant plasmid co-expressing porcine IL-2 and a fusion IL-4/6 gene, when administered with an inactivated PCV2 vaccine, enhanced specific immune parameters, including IgG2a, CD4+, CD8+ T cells, and immune-related gene expression [24]. However, this approach employed a plasmid-nanoparticle delivery system with a monovalent PCV2 vaccine, necessitating investigation of whether the IL-2/IL-4/IL-6 cytokine adjuvant concept can be simplified and adapted for practical PCV2-Mhp bivalent inactivated vaccines. Because the present study uses porcine cytokines in a mouse model, the observed immune effects should be interpreted as preliminary immunogenicity signals rather than definitive evidence of cytokine activity in the target host. Species-specific cytokine-receptor interactions, especially for IL-4, may limit direct translational interpretation and require confirmation in porcine immune cells and piglets.

Manganese-based adjuvants offer additional innate immune stimulation. Mn2+ enhances cGAS-STING signaling and antiviral innate immune activation [25,26,27,28]. Colloidal manganese salt (Mn jelly, MnJ) has demonstrated the capacity to induce both humoral and cellular immune responses, with recent studies continuing to support manganese-based materials as cGAS-STING-associated adjuvants for diverse vaccine antigens [29,30,31]. The MnJ(beta) colloidal manganese adjuvant used in this study consists of water-based manganese nanoparticles containing 5 mg/mL effective manganese with low endotoxin content. We hypothesized that combining porcine IL-2, IL-4, and IL-6 with MnJ(beta) could integrate cytokine-associated adaptive immune modulation with manganese-driven innate immune activation; however, the current design was not powered to prove formal synergy among individual components.

This study therefore evaluated a porcine IL-2/IL-4/IL-6/MnJ(beta) composite adjuvant formulated with PCV2-Mhp bivalent inactivated antigen in an initial mouse model. Mice were selected for first-stage screening because they allow controlled comparison of systemic immunogenicity, hematological tolerability and peripheral lymphocyte phenotypes using well-established immunological reagents at lower cost and with fewer target-species animals than exploratory piglet trials. The primary endpoints were body weight, hematological tolerability and antigen-specific antibody responses; the secondary endpoint was day-56 peripheral blood T- and B-cell phenotyping. The design was intended to prioritize formulations for later piglet studies rather than to demonstrate protection against PCV2 or Mhp infection.

## 2. Materials and Methods

### 2.1. Vaccine Antigen, Cytokines and Colloidal Manganese Adjuvant

The PCV2-Mhp bivalent inactivated antigen was provided by Sichuan Huapai Biotechnology Group Co., Ltd. (Chengdu, China). The antigen contained inactivated PCV2 strain 162 and Mhp strain HP-G, with titers of 10^7.0^ TCID_50_/mL for PCV2 and 10^8.0^ CCU/mL for Mhp prior to inactivation. According to the antigen-preparation records, the PCV2 antigen was inactivated with β-propiolactone at a 1:4000 (*v/v*) ratio at 2–8 °C for 24 h, with mixing every 4 h, followed by hydrolysis at 37 °C for 2 h to terminate inactivation. Complete PCV2 inactivation was verified by immunofluorescence assay, and residual β-propiolactone was checked by HPLC. The Mhp antigen was inactivated by adding 1.0% thimerosal solution prepared by Sichuan Huapai Biotechnology (Group) Co., Ltd. (Chengdu, China) to a final thimerosal concentration of 0.01% and incubating at 2–8 °C for 12 h with intermittent mixing, followed by ultrasonic lysis for 4 min. Complete Mhp inactivation was verified by inoculating 5 mL of inactivated material into 45 mL of Mhp liquid medium prepared by Sichuan Huapai Biotechnology (Group) Co., Ltd. (Chengdu, China) and incubating at 37 °C for 14 days, with subculture onto solid medium during days 5–7; no color change in the liquid medium and no colony growth on solid medium were observed. Porcine IL-2 (652-P2-020/CF), IL-4 (654-P4-025/CF) and IL-6 (686-PI-025/CF) were obtained from R&D Systems (Minneapolis, MN, USA). According to the manufacturer’s specifications, endotoxin levels were <0.10 EU/μg protein for porcine IL-2, <1.0 EU/μg protein for porcine IL-4, and <0.10 EU/μg protein for porcine IL-6, as determined by the LAL method. The cytokine dose of 0.5 µg per cytokine per mouse was selected as an exploratory screening dose based on the manufacturer’s recommended reconstitution range and previous cytokine-adjuvant mouse/piglet studies [24,32].

The colloidal manganese adjuvant (MnJ(β); Qimeng Biotech, Hangzhou, China) was used in this study. This water-based manganese nanoparticle adjuvant contains 5 mg/mL effective manganese with endotoxin levels < 1 EU/mL. The product was stored at 2–8 °C, protected from light. Prior to use, the suspension was vortexed to ensure homogeneous resuspension. Antigen–adjuvant mixtures were prepared fresh by vortexing and administered within 30 min. In the final formulation, MnJ(β) was diluted to deliver 200 μg of manganese per mouse, combined with bivalent antigen and/or cytokines according to experimental groups.

### 2.2. Animals and Experimental Design

Thirty female Kunming mice (6-week-old; *n* = 10/group) were obtained from the Animal Experiment Center of Sichuan University and randomly allocated to three groups. Female mice were used to reduce sex-related variation within this exploratory study and to remain consistent with the institutional animal availability at the time of experimentation; the absence of male mice is acknowledged as a limitation because sex can influence vaccine-induced humoral and cellular responses. All procedures were performed in compliance with institutional animal welfare guidelines (approval code: SYXK-Chuan-2019-172). Mice were maintained under standard laboratory conditions with ad libitum access to food and water.

Immunizations were administered via intramuscular injection in the hind limb, with a booster immunization at week 3 post-primary immunization. Body weights were monitored weekly. Tail vein blood samples were collected at predetermined time points for complete blood count analysis, plasma preparation, antibody detection, and peripheral blood flow cytometry. The experimental design is summarized in Table 1.

### 2.3. Sample Collection and Plasma Preparation

Peripheral blood samples were collected from the tail vein using EDTA-anticoagulated tubes for hematological analysis, plasma preparation, and flow cytometry. For plasma isolation, EDTA-anticoagulated blood was gently inverted several times and centrifuged at 3000 rpm for 10 min at 4 °C. The resulting plasma supernatant was aliquoted and stored at −80 °C until subsequent antibody analysis.

### 2.4. Body-Weight Monitoring and Complete Blood Count

Mouse body weights were recorded weekly from day 0 to day 56 post-primary immunization. Complete blood counts were performed using approximately 50 µL of EDTA-anticoagulated whole blood with a TEK-VET5 veterinary (Tecom Science Corporation, Nanchang, China) automated five-part differential hematology analyzer, following the manufacturer’s protocol. Erythrocyte-, hemoglobin-, and platelet-related parameters served as primary hematological safety indicators, while leukocyte, neutrophil, lymphocyte, and monocyte indices were evaluated as secondary markers of immune activation.

### 2.5. Flow Cytometry Analysis of Peripheral Blood Lymphocyte Subsets

Peripheral blood lymphocytes were analyzed using 100 µL EDTA-anticoagulated blood. Samples were acquired on a BD LSR Fortessa flow cytometer (BD Biosciences, San Jose, CA, USA) and analyzed with FlowJo software (v10.8.1). For T-cell profiling, samples were stained with the following anti-mouse antibodies: CD45 (30-F11, Super Bright 600, eBioscience, Waltham, MA, USA), CD3 (FITC, BD Pharmingen, San Diego, CA, USA), CD4 (GK1.5, eFluor 450, eBioscience, Waltham, MA, USA), CD8a (53-6.7, PerCP-Cyanine5.5, eBioscience, Waltham, MA, USA), CD44 (IM7, APC, eBioscience, Waltham, MA, USA), and CD62L (MEL-14, PE, eBioscience, Waltham, MA, USA). B-cell analysis utilized anti-mouse CD19 (BUV395, BD Biosciences, San Jose, CA, USA), IgM (APC, Thermo Fisher Scientific, Waltham, MA, USA), and IgD (FITC, Thermo Fisher Scientific, Waltham, MA, USA). Antibodies were used at 1 µL per test [33].

Following antibody incubation (30 min, 4 °C in the dark), erythrocytes were lysed with 1 mL 1× RBC lysis buffer (R1010, Solarbio Science & Technology Co., Ltd., Beijing, China) (5 min, room temperature). Cells were then centrifuged (1500 rpm, 5 min, 4 °C), washed twice with PBS (P1020, Solarbio Science & Technology Co., Ltd., Beijing, China) containing 0.5% bovine serum albumin (A8022, Solarbio Science & Technology Co., Ltd., Beijing, China), resuspended in PBS, and fixed with paraformaldehyde (PS1110, olarbio Science & Technology Co., Ltd., Beijing, China) prior to acquisition.

The flow cytometry gating hierarchy was as follows. First, cells were selected using FSC-A versus SSC-A, doublets were excluded using FSC-A/FSC-H and SSC-A/SSC-H, and leukocytes were identified as CD45+ events. T cells were defined as CD45+CD3+ cells and then separated into CD4+ and CD8a+ subsets. CD4+ and CD8a+ T-cell subsets were stratified by CD44 and CD62L expression into naive (CD44low/negativeCD62Lhigh), central memory-like (CD44highCD62Lhigh), and effector memory-like (CD44highCD62Llow/negative) phenotypes. B cells were defined as CD45+CD19+ cells and categorized based on IgM/IgD expression patterns: follicular-like (IgM-IgD+), marginal-zone-like (IgM+IgD+), transitional-like (IgM+IgD-), and IgM-IgD- double negative/activated-like subsets. Because IgG, IgA, GL7 and CD95/FAS were not included, the IgM-IgD- population should be interpreted as a phenotypic approximation rather than definitive class-switched or germinal-center B cells.

### 2.6. Measurement of PCV2-Specific IgG

Plasma PCV2-specific IgG levels were quantified using a modified commercial ELISA kit (Guangzhou Yueyang Biotechnology Co., Ltd., Guangzhou, China), substituting the kit’s secondary antibody with horseradish peroxidase-conjugated goat anti-mouse IgG (Solarbio Science & Technology Co., Ltd., Beijing, China; SE131). Plasma samples were diluted 1:5000 before analysis. The modified detection system was used because the commercial kit was designed for porcine serum, whereas the present study used mouse plasma. To support reproducibility, blank wells, negative mouse plasma and positive vaccinated mouse plasma were included on each plate, and all samples were tested using the same secondary antibody lot and incubation conditions.

### 2.7. Measurement of Mhp-Specific Indirect Hemagglutination Antibody Titers

Mhp-specific antibody titers were determined by indirect hemagglutination assay (IHA). Lyophilized antigen-sensitized erythrocytes (custom-prepared by Sichuan Huapai Biotechnology (Group) Co., Ltd., Chengdu, China; internal reagent code: Mhp-IHA-RBC-20241117; catalog number not applicable) were reconstituted to 2% concentration with 1/15 mol/L PBS (pH 7.2). Test plasma samples were heat-inactivated (56 °C, 30 min) before serial two-fold dilution in a V-bottom 96-well plate containing 25 µL diluent/well. After adding 25 µL of 2% sensitized erythrocytes to each well, plates were mixed (15 s) and incubated (room temperature, 1–2 h). A positive reaction was defined as a diffuse agglutination layer or ring covering the well bottom, whereas a compact button was considered negative. The endpoint titer was defined as the highest plasma dilution demonstrating visible hemagglutination. Because heat inactivation may influence some immunoglobulin classes, IHA results were interpreted primarily as comparative endpoint titers among groups tested under identical conditions.

### 2.8. Statistical Analysis

Data are expressed as mean ± SD. Body weight, complete blood count parameters, and antibody responses were analyzed by two-way repeated-measures ANOVA, with treatment group and time as the two factors, followed by Tukey’s multiple-comparison test. Flow-cytometry data collected on day 56 were analyzed by one-way ANOVA followed by Tukey’s post hoc test. Exact n values are indicated in the corresponding figure legends. Statistical significance was defined as *p* < 0.05. All statistical analyses were performed using GraphPad Prism 7.0.

## 3. Results

### 3.1. The Composite Adjuvant Did Not Suppress Body-Weight Gain

**Figure 1:**
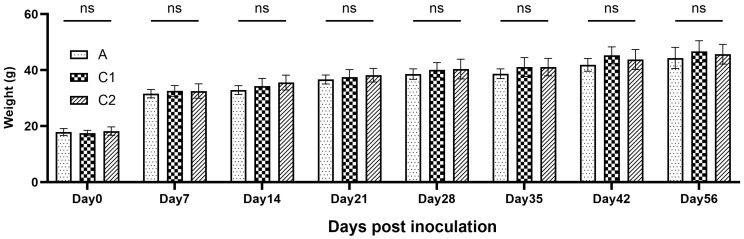
Body-weight changes in mice after immunization. Mice in groups A, C1 and C2 were weighed weekly from day 0 to day 56 after primary immunization. A booster immunization was administered at week 3. Data are presented as mean ± SD (*n* = 10 mice per group at each time point unless otherwise indicated). No significant intergroup differences were detected at the evaluated time points (*p* > 0.05). ns, not significant.

Body weight was monitored throughout the immunization period to evaluate formulation tolerability. All mice exhibited progressive weight gain from day 0 to day 56. As shown in Figure 1, no significant differences were observed among groups A, C1 and C2 at any time point (baseline, primary immunization stage, or post-booster stage; *p* > 0.05), indicating that the IL-2/IL-4/IL-6/MnJ(β) composite adjuvant did not suppress body-weight gain under these experimental conditions.

### 3.2. Hematological Indices Indicated Preliminary Safety with Transient Immune Activation

**Figure 2:**
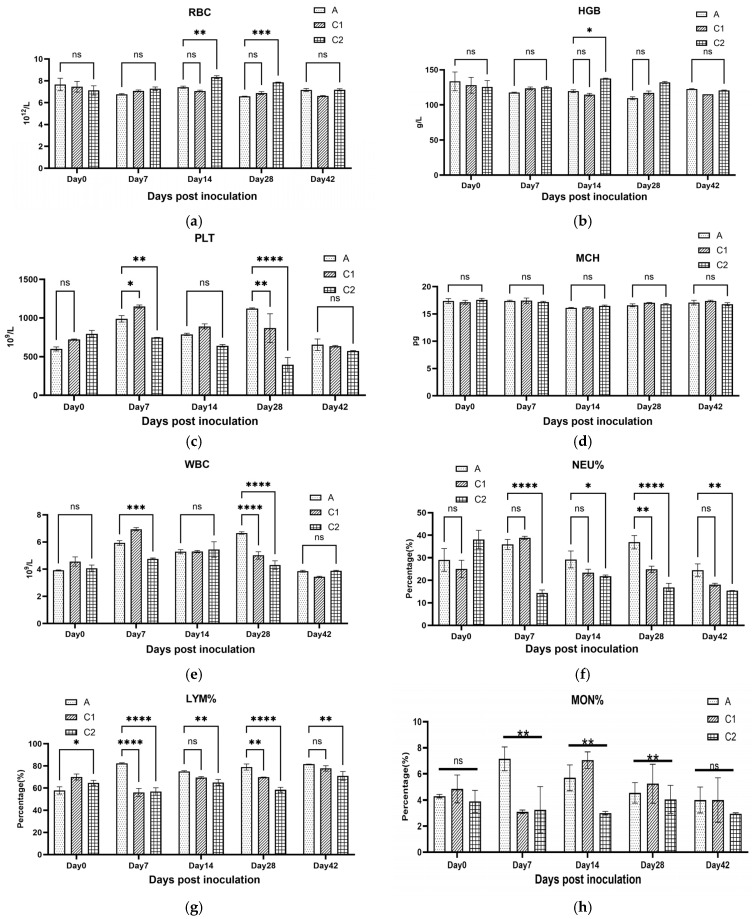
Hematological changes in mice after immunization. (**a**) Erythrocyte number; (**b**) hemoglobin concentration; (**c**) platelet number; (**d**) mean corpuscular hemoglobin; (**e**) white blood cell number; (**f**) neutrophil percentage; (**g**) lymphocyte percentage; (**h**) monocyte percentage. Data are presented as mean ± SD (*n* = 10). Asterisks indicate statistically significant intergroup differences (* *p* < 0.05, ** *p* < 0.01, *** *p* < 0.001, **** *p* < 0.0001); ns, not significant. Leukocyte-related immune parameters increased transiently in group A at selected time points, especially on days 7 and 28 after primary immunization.

Erythrocyte- and platelet-related parameters remained stable across groups throughout the study (Figure 2a–d), with no significant intergroup differences in major safety indices (*p* > 0.05), confirming the formulation’s hematological safety. In contrast, transient adjuvant-associated increases were observed in leukocyte parameters (Figure 2e–h). On days 7 and 28 post-primary immunization, group A showed significantly elevated white blood cell, neutrophil, lymphocyte and monocyte counts compared to PBS control group C2 (*p* < 0.05). By day 28 (7 days post-booster), these parameters in group A remained significantly higher than those in the MnJ-only control group C1 (*p* < 0.05), suggesting time-limited immune activation rather than persistent hematological alterations.

### 3.3. Enhanced B-Cell Activation and Differentiation by the Composite Adjuvant

**Figure 3:**
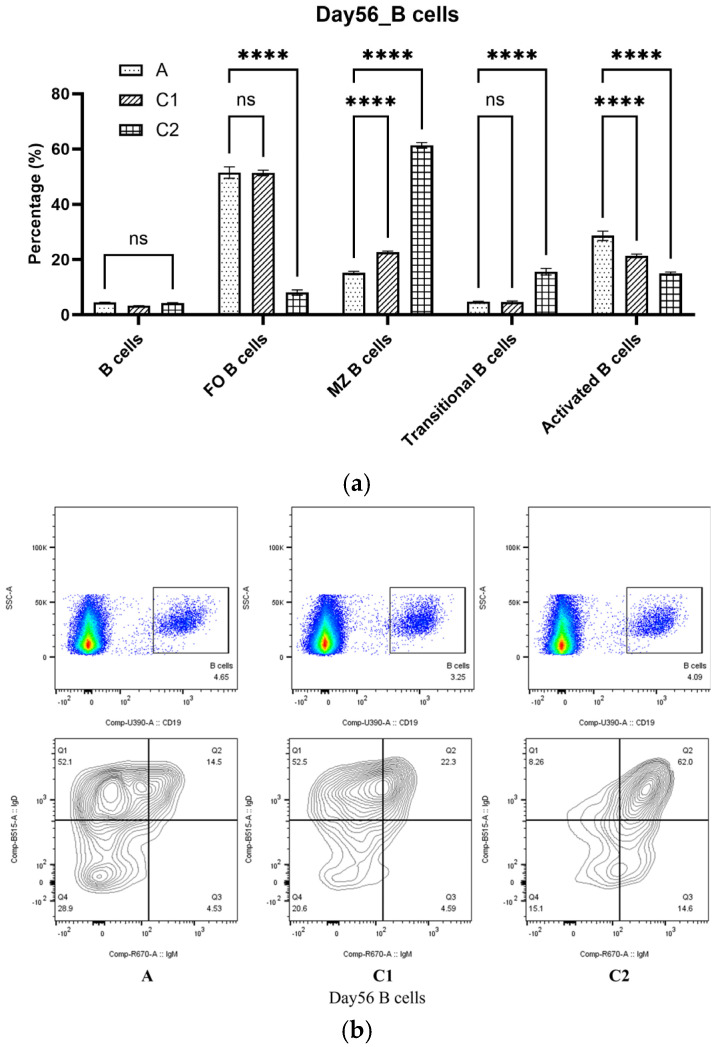
Peripheral blood B-cell subset responses on day 56 after primary immunization. (**a**,**b**) Flow cytometric analysis of CD19+ B−cell subsets classified by IgM and IgD expression, including follicular−like B cells (IgM-/IgD+), marginal−zone−like B cells (IgM+/IgD+), transitional-like B cells (IgM+/IgD-) and IgM-IgD- double−negative/activated−like B cells. Data are presented as mean ± SD (*n* = 10). Group A showed increased IgM-IgD- double-negative/activated−like B cells and follicular−like B cells, together with reduced marginal−zone−like and transitional−like B−cell proportions. ns, not significant; **** *p* < 0.0001.

Day 56 flow cytometry analysis revealed an increase in CD19+IgM-IgD- double-negative/activated-like B cells in group A versus both control groups C1 and C2 (*p* < 0.05). Follicular-like B cells (CD19+IgM-IgD+) were also elevated in group A compared to group C2 (*p* < 0.05), while showing no significant difference versus group C1 (*p* > 0.05). Conversely, group A exhibited significantly lower marginal-zone-like B cells (CD19+IgM+IgD+) than both controls (*p* < 0.05) and reduced transitional-like B cells (CD19+IgM+IgD-) versus group C2 (*p* < 0.05). Because IgG, IgA, GL7 and CD95/FAS were not included, these results indicate shifts in peripheral B-cell phenotypes associated with activation/maturation but do not directly demonstrate germinal-center formation or true immunoglobulin class switching (Figure 3a,b).

### 3.4. Composite Adjuvant Enhanced Memory/Effector T-Cell Responses

**Figure 4:**
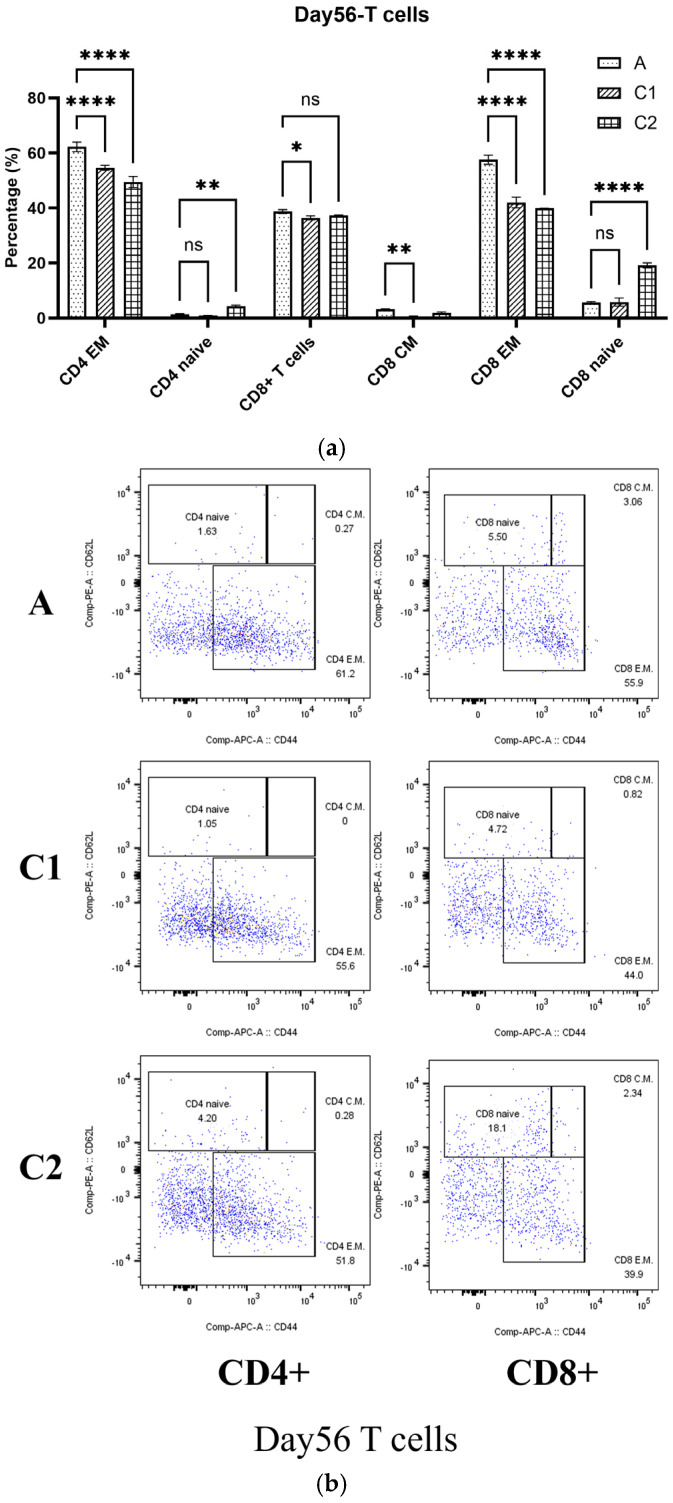
Peripheral blood T−cell subset responses on day 56 after primary immunization. (**a**) CD4+ T−cell memory/effector−associated subsets defined by CD44 and CD62L expression; (**b**) CD8+ T−cell memory/effector−associated subsets defined by CD44 and CD62L expression. T cells were gated as CD45+CD3+ cells. Naive T cells were defined as CD44low/negativeCD62Lhigh, central memory−like T cells as CD44highCD62Lhigh and effector memory−like T cells as CD44highCD62Llow/negative. Data are presented as mean ± SD. (*n* = 10) Group A showed increased effector memory−like CD4+ and CD8+ T−cell subsets compared with control groups. Colors in the representative density plots indicate event density. ns, not significant; * *p* < 0.05, ** *p* < 0.01, **** *p* < 0.0001.

Day 56 T-cell analysis demonstrated significantly higher proportions of effector memory-like CD4+ and CD8+ T cells in group A versus controls (*p* < 0.05; Figure 4a,b). Group A also showed increased CD8+ central memory-like T cells compared to group C1 (*p* < 0.05), while certain CD8+ T-cell indices were elevated versus C1 but comparable to C2 (*p* > 0.05). Naive CD4+ and CD8+ T-cell proportions were lower in group A than C2 (*p* < 0.05), but the lack of a significant difference between A and C1 (*p* > 0.05) indicates that MnJ(beta) alone may have contributed substantially to this shift. Thus, the composite formulation was associated with enhanced effector/memory-like T-cell differentiation compared with PBS, but its incremental effect beyond MnJ(beta) requires confirmation using additional time points and control groups.

### 3.5. Strengthened PCV2-Specific IgG Responses

**Figure 5:**
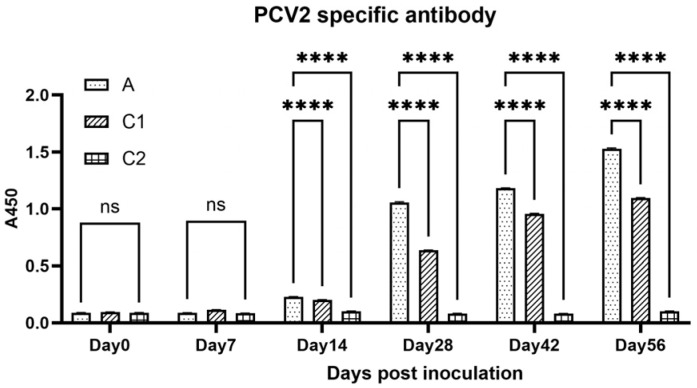
PCV2-specific IgG responses in mouse plasma after immunization. PCV2-specific IgG was measured using a modified commercial ELISA kit with HRP-conjugated goat anti-mouse IgG as the secondary antibody. Data are presented as mean ± SD. *n* = 10. No significant intergroup differences were observed on days 0 and 7 (*p >* 0.05). From day 14 onward, group A showed significantly higher PCV2-specific IgG levels than groups C1 and C2 (*p <* 0.05). ns, not significant; **** *p* < 0.0001.

PCV2-specific IgG levels were initially low and comparable across groups (days 0–7; *p* > 0.05). From day 14 onward, vaccinated groups showed increasing antibody responses, with group A demonstrating significantly higher PCV2-specific IgG levels than both control groups during follow-up (*p* < 0.05; Figure 5). These data indicate that the IL-2/IL-4/IL-6/MnJ(beta) formulation enhanced the magnitude of PCV2-specific IgG responses and maintained elevation during the 56-day observation period compared with MnJ(beta) alone. PCV2-specific IgG responses increased progressively after immunization. In group A, the OD450 values increased from 0.088 ± 0.003 at day 0 to 0.230 ± 0.001 on day 14, 1.057 ± 0.006 on day 28, 1.181 ± 0.005 on day 42, and 1.532 ± 0.006 on day 56. In comparison, group C1 reached 1.095 ± 0.004 at day 56, whereas the PBS control group C2 remained at a low background level of 0.102 ± 0.002. Thus, at day 56, the composite IL-2/IL-4/IL-6/MnJ(beta) formulation increased PCV2-specific IgG by approximately 1.40-fold compared with MnJ(beta)-adjuvanted antigen alone and by 15.09-fold compared with PBS.

### 3.6. The Composite Adjuvant Improved Late Maintenance of Mhp-Specific Antibody Titers

**Figure 6:**
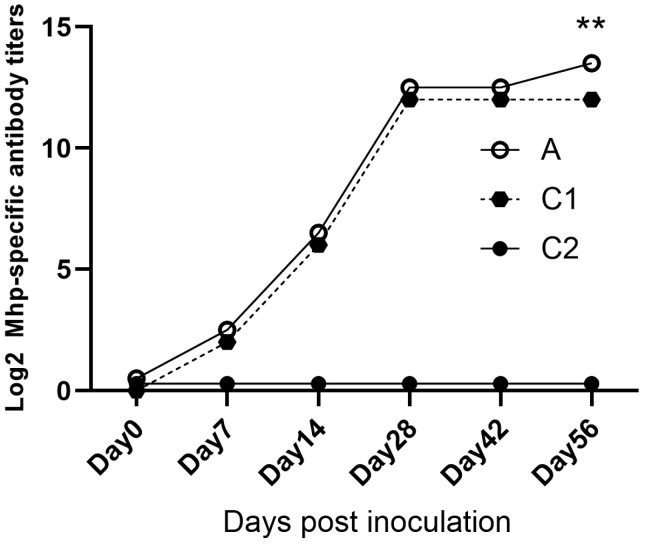
Mhp-specific indirect hemagglutination antibody titers in mouse plasma after immunization. The endpoint titer was defined as the highest plasma dilution showing a positive hemagglutination reaction. Data are presented as mean ± SD or endpoint titers as appropriate, *n* = 3. Group A maintained higher late-stage Mhp-specific IHA titers, and the day-56 titer was significantly higher in group A than in groups C1 and C2 (*p <* 0.05). ** *p* < 0.01.

Both vaccinated groups showed rising Mhp-specific IHA titers post-immunization, while PBS controls remained low. Although groups A and C1 showed comparable early responses, group A maintained superior antibody levels during late-stage observation. By day 56, group A exhibited significantly higher Mhp-specific IHA titers than both control groups (*p* < 0.05; Figure 6), indicating improved maintenance of Mhp-specific antibody responses within the 56-day observation period. Mhp-specific IHA titers showed a similar booster-associated increase. The log2 endpoint titers in group A increased from 2.00 ± 0.00 on day 7 to 6.00 ± 0.00 on day 14 and 12.00 ± 0.00 on days 28 and 42, before increasing further to 13.00 ± 0.00 on day 56, corresponding to a GMT of 1:8192. Group C1 reached a log2 endpoint titer of 12.00 ± 0.00 on day 56, corresponding to a GMT of 1:4096, whereas the PBS control group remained negative throughout the observation period. These data indicate that the composite cytokine/MnJ(beta) formulation enhanced late PCV2-specific IgG magnitude and maintained a one-dilution higher Mhp-specific IHA endpoint titer than MnJ(beta)-adjuvanted antigen alone at day 56.

## 4. Discussion

Cytokines are pivotal regulators of immune responses, modulating the balance between humoral and cellular immunity. IL-2 promotes T-cell proliferation and cytotoxic function, IL-4 facilitates B-cell maturation and antibody class switching, and IL-6 drives B-cell differentiation and inflammatory signaling [19,20,21]. Previous PCV2 studies in piglets demonstrated that co-delivery of porcine IL-2 with IL-4/6 enhanced CD4+ and CD8+ T-cell responses while modulating immune-related gene expression [24]. The current findings are directionally consistent with these functions, as evidenced by increased effector memory-like T-cell subsets and CD19+IgM-IgD- double-negative/activated-like B cells in the composite adjuvant group. Nevertheless, because porcine cytokines were administered to mice, this interpretation must be cautious. The study does not establish direct receptor-mediated bioactivity of each porcine cytokine in murine cells, and species-specific cytokine-receptor interactions may contribute to differences between the murine screening model and piglets.

Available product bioactivity data support the responsiveness of mouse-derived cell lines to porcine IL-2 and IL-6, whereas direct murine bioactivity of porcine IL-4 was not verified in this study; therefore, the present data should be interpreted as composite-formulation screening results rather than definitive evidence that each porcine cytokine independently exerted direct receptor-mediated effects in mice.

Manganese potentiates cGAS-STING signaling, enhances antigen uptake/presentation, and supports germinal center and cellular immune responses [25,26]. MnJ has demonstrated broad adjuvant activity across experimental systems, with emerging evidence supporting manganese-based formulations as versatile adjuvant platforms. Here, MnJ(β) functions not merely as a carrier but as an active immunostimulatory component. Its combination with IL-2, IL-4 and IL-6 creates a dual-phase adjuvant effect: manganese-driven innate immune activation during early vaccination, followed by cytokine-mediated T- and B-cell differentiation in the adaptive phase. However, because antigen-alone, cytokine-only and antigen-plus-cytokine control groups were not included, the present data cannot quantify independent component effects or prove formal synergy.

The safety observations in this murine model should be interpreted as preliminary tolerability data rather than a complete toxicological assessment. Although the steady weight gain and absence of sustained erythrocyte/hemoglobin suppression were observed throughout the study period, which corroborate prior safety reports for cytokine adjuvants [32] and align with established safety profiles of MnJ-based formulations [28], the transient leukocyte increases likely reflected immune activation by the inoculation. However, injection site pathology, serum biochemical indices of liver/kidney function, histopathology of lymphoid and major organs, and manganese tissue residue measurements were not performed. Therefore, additional systemic safety and biodistribution studies are required before target-species application.

For PCV2-Mhp vaccines, protective performance relies on coordinated humoral and cellular immunity. Current commercial and experimental vaccines demonstrate that reduced viremia, pathogen shedding, and lung lesions—along with improved growth performance—depend on this immune coordination [13,14,15,16,17,18]. While earlier PCV2 studies in piglets showed comparable total antibody levels between cytokine-adjuvanted and control groups (with enhanced IgG2a and cellular immunity) [24], our composite adjuvant induced earlier PCV2-specific IgG responses and maintained Mhp-specific antibody titers during the observation. This discrepancy may reflect differences in antigen composition, species, delivery format, or MnJ(β)-mediated innate immune activation. Importantly, the present study did not measure PCV2/Mhp challenge protection, viral clearance, bacterial clearance, lung lesions, mucosal IgA or neutralizing antibodies; therefore, no conclusion can be made regarding protective efficacy.

Flow cytometry data further support this interpretation. Recent PCV2 vaccine studies highlight the protective role of vaccine-induced T-cell responses, particularly multifunctional and memory T-cell populations [17,18]. Similarly, Mhp vaccine research emphasizes the need for comprehensive protection beyond traditional bacterins, requiring robust local, innate and adaptive immunity [11,12,34]. Our findings of expanded effector memory CD4+/CD8+ T-cell subsets suggest enhanced recall capacity, while increased activated/class-switched B cells with reduced transitional populations indicate progression toward mature, antigen-experienced phenotypes, which confirms the elevation of specific humoral immunity.

## 5. Conclusions

In summary, the IL-2/IL-4/IL-6/MnJ(beta) formulation extends previous cytokine adjuvant strategies by combining cytokine-associated adaptive immune modulation with manganese-mediated innate immune stimulation. This composite adjuvant enhanced antibody responses while promoting peripheral immune-cell activation and effector/memory-like differentiation in a PCV2-Mhp bivalent antigen mouse model, providing a rationale for further validation in porcine immune cells and piglet vaccination/challenge studies.

## 6. Limitations

Several limitations should be considered. First, mice are not the target host for PCV2-Mhp vaccines and cannot fully recapitulate porcine respiratory infection or mucosal immunity. Second, porcine cytokines were used in mice, and the cross-species bioactivity of each cytokine was not directly validated despite the cross-reaction of species. Third, the study did not include PCV2 or Mhp challenge, viral/bacterial clearance assays, lung-lesion scoring, neutralizing antibody assays, IgG subclass analysis, or mucosal IgA measurements. Fourth, antigen-alone, cytokine-only and antigen-plus-cytokine control groups were not included, preventing formal evaluation of individual component effects or synergy. Fifth, only female mice were used. Sixth, flow cytometry was performed only on day 56, limiting the kinetic interpretation of lymphocyte activation. Seventh, safety assessment was limited to body weight and hematological indices; injection-site pathology, serum biochemistry, histopathology and manganese tissue-residue analyses were not performed. Finally, the 56-day observation period is insufficient to establish long-term durability of immune memory. These limitations justify subsequent piglet studies with expanded controls, longitudinal immune profiling and challenge-protection endpoints.

## Figures and Tables

**Table 1:** Experimental grouping and immunization regimen.

Group	Treatment
A	0.1 mL PCV2-Mhp bivalent inactivated antigen + porcine IL-2, IL-4 and IL-6 (0.5 µg each per mouse) + MnJ(beta) colloidal manganese adjuvant (200 µg effective Mn per mouse)
C1	0.1 mL PCV2-Mhp bivalent inactivated antigen + MnJ(beta) colloidal manganese adjuvant (200 µg effective Mn per mouse)
C2	0.2 mL phosphate-buffered saline (PBS)

## Data Availability

The datasets generated or analyzed during the current study are not publicly available due to commercial confidentiality but are available from the corresponding author on reasonable request.

## Abbreviations

The following abbreviations are used in this manuscript:

PCV2	Porcine circovirus type 2
Mhp	Mycoplasma hyopneumoniae
PRDC	Porcine respiratory disease complex
IL	Interleukin
MnJ	Colloidal manganese adjuvant
CBC	Complete blood count
IHA	Indirect hemagglutination assay
ELISA	Enzyme-linked immunosorbent assay
cGAS-STING	Cyclic GMP-AMP synthase-stimulator of interferon genes pathway
PBS	Phosphate-buffered saline
